# Individualized Tailor-Made Dietetic Intervention Program at Schools Enhances Eating Behaviors and Dietary Habits in Obese Hispanic Children of Low Socioeconomic Status

**DOI:** 10.1155/2014/484905

**Published:** 2014-01-30

**Authors:** Leticia Elizondo-Montemayor, Diana Moreno-Sànchez, Norma G. Gutierrez, Fabiola Monsivais-Rodriguez, Ubaldo Martinez, Ana C. Lamadrid-Zertuche, Martin M. Hernandez-Torre

**Affiliations:** ^1^Clinical Nutrition and Obesity Research Center, School of Medicine and Health Sciences, Tecnológico de Monterrey, Avenue Morones Prieto 3000 Pte., Col. Los Doctores, 64710 Monterrey, NL, Mexico; ^2^Tecnológico de Monterrey, Avenue Morones Prieto 3000 Pte., Col. Los Doctores, 64710 Monterrey, NL, Mexico

## Abstract

Hispanic children and those from low-socioeconomic status are predisposed to unhealthy eating habits and obesity. *Aim*. to implement an individualized, face-to-face, parent supported, and school-partnership dietetic intervention to promote healthy eating habits and decrease body mass index. Prospective school year dietetic intervention of 101 obese, Hispanic, low-socioeconomic school-age children representative of Monterrey, Mexico, consisted of anthropometrics, dietetic assessment, energy-restriction tailor-made daily menus, and parental education every three weeks. Student's *t*-test was used for means comparison. A significant decrease was found in body mass index percentile (96.43 ± 3.32 to 93.42 ± 8.12/P = 0.00) and energy intake/day of −755.7 kcal/day (P = 0.00). Among other energy dense foods with significant decline in servings/day and servings/week were processed meats (3.13 ± 1.43 to 2.19 ± 1.04/P = 0.00 and 5.60 ± 1.75 to 4.37 ± 2.10/P = 0.00, resp.), saturated fat (1.47 ± 1.08 to 0.78 ± 0.79/P = 0.00 and 2.19 ± 2.18 to 1.1 ± 1.36/P = 0.00), sweetened beverages (2.79 ± 1.99 to 1.42 ± 1.21 and 6.21 ± 1.72 to 3.89 ± 2.80/P = 0.00), and desserts and refined-grain bakery (1.99 ± 1.54 to 1.32 ± 1.59 and 2.85 ± 2.54 to 1.57 ± 2.20/P = 0.00). There was a significant increase in servings/day and servings/week of water (2.98 ± 2.02 to 4.91 ± 2.37 and 6.62 ± 2.03 to 6.87 ± 0.91/P = 0.00, resp.) and nutrient dense foods such as fruits (1.31 ± 0.89 to 1.66 ± 0.96 and 3.34 ± 2.24 to 4.28 ± 2.43/P = 0.00) and fish and poultry (3.76 ± 2.15 to 4.54 ± 2.25/P = 0.00). This intervention created healthy eating habits and decreased body mass index in a high risk population. Trial registration number: NCT01925976.

## 1. Introduction

Obesity in children is a rapidly expanding disease across the world [1]. Currently, with a 32.8 percent of adult obesity rate, Mexico is just inches past the 31.8 percent obesity rate in the United States, according to a study released on June 4th, 2013 by the United Nations Food and Agricultural Organization. Mexico has one of the highest rates of obesity in the world [2]. However, the last national survey made in Mexico, in 2012, states that for adults, the combined prevalence of obesity and overweight in the country is 64.9%, and in school-aged children it reaches 34.4%, with a gender difference of 36.9% for boys and 32% for girls [3]. Childhood obesity carries great health risks for children; it is associated with insulin resistance and increased risk of developing cardiovascular disease, type 2 diabetes, hypertension, dyslipidemia, long-term vascular complications, and the metabolic syndrome [1, 4]. Furthermore, overweight and obese children are likely to become obese as adults and to develop cardiovascular and metabolic complications at a younger age [1].

Ethnicity also plays a role in predisposition to obesity. It has been demonstrated that Hispanic children and adolescents have greater proportion of visceral fat and obesity [5, 6] and are likely to have less healthy eating habits [7, 8] than those that are not Hispanic. As well, socioeconomic status contributes to obesity rates and less healthy eating habits. It has been established that children from low socioeconomic status (LSE status) are also less likely to have healthy eating habits than those from higher income status, since it has been shown that they consume more energy dense (ED) and nutrient-poor diets [9, 10], which makes them even more prone to overweight and obesity [9, 11, 12].

The appropriate approach to decrease overweight and obesity prevalence, according to the Academy of Nutrition and Dietetics [13], the American Heart Association [1], and the American Academy of Pediatrics [14] is through change in dietary habits and physical activity. Studies find that parental food choices influence children's food preferences, dietary habits, and nutrient intakes [1, 15, 16], so parental education regarding healthy eating is of the upmost importance. National associations [13, 14] recommended that health providers should work with communities and schools to help individuals modify their health habits as part of their efforts to control obesity. Although some school-based interventions directed at curriculum modifications or changing school policies have had positive effects on improving healthy eating habits [16], some others have not [17, 18]. Many of these school-based studies had some significant limitations; they either neglected or poorly addressed the importance of parental support, or what children ate outside of school, or they were short-term and they were not carried out on an individualized basis. Currently, there is only limited available evidence about effective strategies to manage childhood obesity [1]; therefore, innovative dietetic interventions are needed. Few comprehensive dietetic intervention programs have been carried out with obese Hispanic children from LSE status, and no studies have used an individualized, tailor-made approach.

Therefore, the aims of this study were to implement an innovative, comprehensive, individualized, face-to-face with the registered dietitian, at school, and parent supported, school year dietetic intervention program aimed at promoting healthy eating in obese Hispanic children from LSE status to (1) decrease body mass index percentiles (BMI), (2) examine the modifications in energy intake, (3) identify variations in the consumption of macronutrients, (4) determine changes in the contribution of individual food groups to eating habits, and (5) detect gender differences regarding eating habits.

## 2. Materials and Methods

### 2.1. Study Population

This was a prospective interventional study. An open invitation was made to children ages 5–12 years from eight public schools of LSE status, representative of Monterrey, the second largest city in Mexico, to screen for overweight and obesity. From a total of 4300 children in these schools, 1300 children were randomly selected for screening for overweight and obesity using BMI percentiles with an error <1.4% for the sample. Overweight/obesity was found in 451 children, who were all invited to participate in the dietetic intervention program at no cost. From these, 125 children accepted to participate and 101 completed the school-year dietetic intervention program. The rest of the overweight/obese children's parents were not interested in the intervention program, or did not meet all of the inclusion criteria, but most of them were simply not concerned. Some of the reasons why 24 children dropped out of the program were due to change of school, lack of parent or caregiver attendance during the visit, or absence to school during some of the consultations. Inclusion criteria were attendance from first to sixth grade; ages 6–12 years; BMI ≥ 85th percentile for age and sex; Hispanic; both parents Hispanics; LSE status; and signed consent from both parents/caregivers and active assent from children. Exclusion criteria were disapproval by the children's physician due to any at-risk medical condition known by the parents. Approvals by the Ethics and Research Committees of the School of Medicine Tecnológico de Monterrey and by the State Education Authorities were obtained. Participants did not receive compensation for participating.

### 2.2. Clinical Evaluation

Based on the World Health Organization and the American Academy of Pediatrics criteria [14], overweight was considered as BMI ≥ 85th and <95th percentiles and obesity as ≥95th percentile according to age and sex.

Anthropometric measurements were performed in all participants at each school. Standing height was determined to the nearest 0.5 cm (portable Seca stadiometer, North America) and weight to the nearest 0.1 kg while children wore light clothing and no socks or shoes (TANITA TBF 300 scale, Arlington, Illinois). Waist circumference was measured to the nearest 0.1 cm at the level of the umbilicus with a flexible fiberglass tape while the subjects were standing, after gently exhaling, and with no clothing on the area. BMI was calculated by weight (kg) divided by the square of height (m). Measurements were performed by the same three trained registered dietitians (RD) on all children to control the interobserver variability.

### 2.3. Dietetic Intervention

The principal strategies for the change in energy and food groups consumption were dietary modifications for the children and parental support, as recommended by national associations [1, 13, 14, 19]. Dietetic intervention was given individually by a RD for every child at each school, every three weeks, for a total of 13 visits during the school-year. Each child left the classroom to attend the 45-minute nutrition counseling and was accompanied by a parent or caregiver. Each child and parent or caregiver was seen by the same RD throughout the school-year to favor compliance to the plan and to avoid interexaminer bias. Each session was conducted by the RD in terms that children and parents could understand clearly. Each nutrition counseling session consisted of (1) anthropometric assessment; (2) dietetic assessment by means of 24-hour diet recalls, a standardized food frequency questionnaire that included Mexican foods, and food replicas to aid in estimation of portion sizes; (3) individualized energy restriction and balanced macronutrient dietary planning; (4) provision of structured, tailor-made daily menus and meals for the next three weeks for each child; and (5) information given to parents/caregivers about healthy food, eating practices, and portion sizes. Attendance of the parent/caregiver was mandatory to help answer the 24-hour recalls and to assure commitment to follow the dietary recommendations at home. School partnership consisted in giving notification to parents and children one and two days before the visit, letting the child leave the classroom and attend the nutrition program in a room provided by the school. After each visit, the RD recorded the information into the software (NutriKcal VO software, Consinfo, S.C., DF, Mexico), which determined energy intake and diet composition. Entry of data into the software was completed by means of serving/day and days/week including the weekends.

Diet composition (macronutrients) was based on the most recent dietary recommended intake for children ages 6–12 [19]: 25%–35% of total calories from fat, 45%–65% from carbohydrates, and 10%–30% from protein. Additionally, according to the American Heart Association and the National Cholesterol Education Program recommendations for children or adolescents included a variety of foods that are low in saturated fat (<10% kcal), no trans fat, and cholesterol <300 mg/day. The RDs promoted age appropriate serving sizes, including approximately ≥5 servings of fruit and vegetables, ≥3 servings of low fat milk or dairy products, and ≥6 servings of whole-grain products per day; increase of dietary fiber; and reduction of salt intake [20]. As well, children were advised to avoid overconsumption of ED and nutrient-poor foods and beverages [13].

Reduction in calorie intake was approached following the recommendations of the American Heart Association in which children >4 years old with a BMI ≥ 85th percentile may achieve BMI percentile reductions to <85th percentile with weight maintenance during linear growth [1]. As advised [14], progressive restriction of 150–900 calories from actual intake throughout the school-year was recommended so that obese children with BMI > 95th percentile could lose gradually 0.5 kilogram/month and those with BMI > 99th percentile could lose a maximum of 0.9 kilogram/week.

### 2.4. Statistical Methods

MINITAB version 16 (Minitab Inc., State College, PA, USA) was used to analyze the differences between anthropometric parameters and nutrient intake values at baseline and end of intervention; Microsoft Excel 2007 (Microsoft Corp., Redmond, WA, USA) was used to incorporate the input of data. The results were expressed as mean ± standard deviation (s.d.) and their corresponding 95% confidence intervals (CI). Comparisons between groups for dependent variables were made using paired Student's *t*-test for means. The mean comparisons between gender groups were determined using *t*-test for independent samples. All tests were interpreted based on two-tailed hypothesis. The significance level was set at 0.05 in all cases. The statistical power was >0.80 for all statistical tests involved in this study's analysis.

## 3. Results

Table 1 shows baseline and end of the school-year dietetic intervention values. BMI percentile fell significantly by a mean difference of −3.0  (*P* = 0.00), from 96.43 to 93.42 (−4.27, −1.75; 95% CI). There was a significant decrease of −755.7 calories (−907.4, −604.1; 95% CI; *P* = 0.00) of the total energy intake/day. As well, there was a significant calorie reduction of all macronutrients consumption (*P* = 0.00). Although girls showed greater reduction in anthropometric parameters, total energy consumption/day, and macronutrients intake/day compared to boys, there was no significant gender difference (Table 2). Children maintained the recommended dietary composition of the total energy intake/day and by the end of the intervention: fat 25%–35% (mean: 24.52 ± 7.96); carbohydrates 45%–65% (mean: 58.13 ± 9.101); and protein 15%–30% (mean: 17.35 ± 3.90) (Table 1).

Concerning daily servings and servings/week of the different food groups, snacks, fast foods, and beverages, Table 3 shows the baseline and end of the school-year dietetic intervention values. There was a significant increase in servings/day of fruits (1.31 to 1.66) (*P* = 0.00), and specially of water (2.98 to 4.91) (*P* = 0.00), while there was a considerable decrease of processed meats (3.13 to 2.19) (*P* = 0.00), oils (3.76 to 1.39) (*P* = 0.00), sugar sweetened beverages (2.79 to 1.42) (*P* = 0.00), saturated fat (1.47 to 0.78) (*P* = 0.00), desserts and refined-grain bakery products (1.99 to 1.32) (*P* = 0.00), whole fat milk (1.72 to 1.23) (*P* = 0.00), sweets (1.85 to 1.35) (*P* = 0.00), fast food meals (0.96 to 0.63) (*P* = 0.02), and chips and french fries (1.13 to 0.68) (*P* = 0.00). There was a significant increase in the number of days/week consumption of fish and poultry (3.76 to 4.54) (*P* = 0.00) and no difference in whole grains intake.

Regarding the number of days/week intake of foods, there was a significant increase in fruits (3.34 to 4.28) (*P* = 0.00), fish and poultry (3.76 to 4.54) (*P* = 0.00), and water consumption (6.62 to 6.87) (*P* = 0.00), while there was a significant decrease in the intake of processed meats (5.60 to 4.37) (*P* = 0.00), saturated fat (2.19 to 1.10) (*P* = 0.00), sweets (3.02 to 2.20) (*P* = 0.00), sugar sweetened beverages (6.21 to 3.89) (*P* = 0.00), chips and fries (2.60 to 1.36) (*P* = 0.00), and desserts and refined-grain bakery products (2.85 to 2.54) (*P* = 0.00). Although in general girls showed more favorable healthy eating changes in days/week intake compared to boys, there was no significant gender difference, except for oils (Table 4).

## 4. Discussion

According to the Academy of Nutrition and Dietetics, the number of American children who are overweight has more than tripled among 6- to 11-year-old children, which has major health consequences [13]. It is well recognized that the obesity epidemic in children is due in part to the consumption of unhealthy foods and drinks and low physical activity [1]. This is the first study in Mexico to evaluate the effects of an innovative one school-year, face-to-face, individualized, at school, and parent supported dietetic intervention on eating habits of Hispanic, LSE status, school-aged children. Findings were noteworthy in favor of modification towards healthier eating habits. A significant decrease was found between baseline and end-of-year intervention in BMI percentile by a mean difference of −3.0 (*P* = 0.00) as well as of energy intake/day, while children maintained their macronutrient consumption within the recommended ranges. Reduction of calorie intake in this population is of impact, as it is well known that the most important factor in obesity development is an imbalance between calorie intake and calorie expenditure.

Diverse studies have found an association between the consumption of ED foods and obesity [8, 21] and higher fat mass [22] in children. On the other hand, a pattern characterized by ND foods was associated with smaller gains in BMI [23]. The findings in this study clearly indicate a significant decline in servings/day and servings/week consumption of processed meats, oils, saturated fat, sweetened beverages, desserts, refined-grain bakery products, whole fat milk, sweets, fast food meals, chips and fries, and all ED foods. On the other hand, children improved further their eating habits by increasing significantly the intake of water and that of fruits, fish, and poultry, which are ND foods. Although the increase in fruits and vegetables is small, it is still significant. These findings produced an impact on the well-being of the children and were in accordance with national recommendations to improve fruit and vegetable consumption and decrease intake of fats and added sugars [20], as well as to favor a low consumption of ED foods and an increased consumption of ND foods [13, 24, 25].

The favorable impact on health habits in this interventional study is even more relevant, considering the fact that our population is from LSE status, and thus, at higher risk, as it is well recognized that downward mobility or stable LSE status has been associated with greater adiposity evaluated by BMI, waist circumference, and triceps skin fold [11]. This link has also been found in cross-sectional studies [10] and in longitudinal ones that even associate LSE status in childhood with adult obesity [26]. Even more, the prevalence of childhood obesity almost doubled across levels of household income [12]. In addition, LSE status has also been associated with poor and unhealthy eating [10, 27] such as low vegetable consumption and high intake of fried foods [9] and consumption of ED fast foods [27]. Thus, the fact that our at-risk population changed their dietary consumption of all food groups in a favorable way is important not only for the reduction in their actual BMI percentiles, but it may also contribute to their health status and in prevention of cardiometabolic chronic diseases such as insulin resistance, diabetes mellitus, dyslipidemias, and the metabolic syndrome in the near future.

Several studies have demonstrated that Hispanic children and adolescents, such as our studied population, have a greater proportion of obesity, visceral fat, and type 2 diabetes [5, 6], as well as greater morbidity and mortality [28]. Besides, research has shown that Hispanic children have a tendency towards nonhealthy eating habits, such as low consumption of fruits, vegetables, and milk and higher intake of saturated fat, fries, fast food, and high ED foods such as refined-grain bakery products [7]. In a study conducted on school-age Mexican children, patterns characterized by high intakes of ED foods were associated with a higher risk of overweight/obesity [8]. These facts highlight the importance of our dietetic intervention, which achieved an improvement towards healthy eating habits in this at-risk Hispanic population.

However, although some school-based interventions in other countries that were directed at curriculum modifications or changing school policies, or the selling or provision of food within the schools have had positive effects on improving healthy eating habits [16], some others have not [17, 18] because they were short-term, they either neglected or poorly addressed what children ate outside of school or the importance of family support [29]. Furthermore, the dietetic interventions have not been carried out on an individualized basis, as ours has been. Our dietetic intervention was carried out at the schools as a partnership. The school administrative personnel and the teachers reminded children of the next nutrition consultation both verbally and by written notification. On the visit day, children were called out of the classroom for their individual consultations with the Rds. This school support facilitated enormously the adherence to the dietetic intervention.

It is also well known that parents influence food preferences and eating behaviors of children, as demonstrated in various studies [7, 15, 29] and as stated by national associations [13, 14]. Previous studies have shown an association between more availability of unhealthy food products, more permissiveness from parents and less healthy food choices among Hispanic children and adolescents [7, 15]. Thus, nutrition education for Hispanic parents of LSE status seems especially important for improving children's diets. Favorable results of this study might also be due to the fact that parent/caregiver presence was mandatory at the time of the consultations with the RD and they were instructed regarding nutrition education and healthy eating habits for the whole family and how to deal with the food preferences of their particular child participating in the study.

The study had some limitations, though. Voluntary participation in the study and the relatively small sample may have influenced our results and may not be representative of the general population. Not having a control group could have limited the impact of this intervention. The children and their parents/caregivers were likely to be highly motivated and thus the study could be biased in this regard.

Nevertheless, the study had several strengths. Factors contributing to the changes in healthier eating habits and the decrease in BMI percentiles were the parental support, the school partnership, the year-long intervention, the face-to-face consultations with the RDs, and the individualized tailor-made menus that the RDs planned for each child. A long-term follow-up plan is currently being structured to be able to measure the success of the dietary intervention.

## 5. Conclusions

Our findings in obese, Hispanic, LSE status children demonstrating a change in dietary patterns towards healthier eating habits, contribute to our understanding that maybe the strategy we followed is needed to help overcome obesity and to move towards healthier eating habits. To the best of our knowledge, this is the first initiative of its kind that we found in literature search. Although the solution is complex, intervention strategies that focus on individual children's attitudes and parental behavior should aim at creating individual behavior changes. Having a home and school environment with healthy eating behaviors is recommended to encourage children to maintain these new eating habits in the long term.

## Figures and Tables

**Table 1:** Comparison of anthropometric parameters and nutrient intake at baseline and end of dietetic intervention.

Variable	Sample size *n* = 101				
	Baseline	End of intervention	Mean difference	95% CI*	*P* value
BMI percentile	96.43 ± 3.32	93.42 ± 8.12	−3.0	(−4.27, −1.75)	0.00
Waist circumference (cm)	83.29 ± 9.17	82.82 ± 9.88	−0.5	(−1.54, 0.61)	0.39
Total energy (kcal/day)	2506.9 ± 700.6	1751 ± 468.2	−755.7	(−907.4, −604.1)	0.00
Carbohydrates					
Energy (kcal/day of carbohydrates)	1366 ± 438.6	1019.1 ± 351.7	−346.8	(−449.2, −244.5)	0.00
Grams (grams)	348.2 ± 149.7	256.3 ± 85.1	−91.9	(−125.0, −58.8)	0.00
Percent calories (%)	54.4 ± 9.132	58.13 ± 9.101	3.7	(1.38, 6.08)	0.00
Protein					
Energy (kcal/day of protein)	400.3 ± 117.9	299.5 ± 87.6	−100.7	(126.9, −74.5)	0.00
Grams (grams)	100.09 ± 29.4	74.39 ± 22.03	−25.7	(−32.47, −18.93)	0.00
Percent calories (%)	16.37 ± 4.14	17.348 ± 3.90	0.99	(0.070, 1.90)	0.03
Fat					
Energy (kcal/day of fat)	713.5 ± 305.0	418.4 ± 184.2	−295.1	(−356.3, −233.9)	0.00
Grams (grams)	79.29 ± 33.88	47.35 ± 19.30	−31.9	(−38.73, −25.13)	0.00
Percent calories (%)	28.032 ± 7.66	24.515 ± 7.96	−3.5	(−5.48, −1.54)	0.00

Data represent mean ± standard deviation. *Confidence interval for the mean difference. The significance level used in paired *t*-test was 0.05.

**Table 2:** Gender comparison of the mean difference of anthropometric parameters and nutrient intake after dietetic intervention.

Variable	Boys (*n* = 55)	Girls (*n* = 46)	95% CI*	*P* value
BMI percentile	−2.61 ± 4.32	−3.49 ± 8.23	(−1.65, 3.43)	0.49
Waist circumference (cm)	−1.19 ± 4.96	0.4 ± 5.96	(3.74, 0.57)	0.15
Total energy (kcal/day)	−649 ± 693	−883 ± 839	(−69, 536)	0.13
Carbohydrates				
Energy (kcal/day of carbohydrates)	−246 ± 455	−467 ± 566	(20, 423)	0.03
Grams (grams)	−74 ± 188	−114 ± 139	(−26.5, 106.1)	0.24
Percent calories (%)	4.9 ± 12.2	2.4 ± 11.5	(−2.18, 7.24)	0.29
Protein				
Energy (kcal/day of protein)	−92 ± 129	−112 ± 138	(−32.6, 72.9)	0.45
Grams (grams)	−23.8 ± 34.3	−28 ± 34.6	(−9.47, 17.82)	0.55
Percent calories (%)	0.56 ± 4.56	1.49 ± 4.72	(−2.76, 0.906)	0.32
Fat				
Energy (kcal/day of fat)	−291 ± 328	−300 ± 291	(−11.3, 132.8)	0.88
Grams (grams)	−31.6 ± 36.2	−32.4 ± 32.7	(−12.91, 14.53)	0.91
Percent calories (%)	−4.1 ± 10.4	−2.86 ± 9.58	(−5.18, 2.77)	0.55

Data represent mean ± standard deviation. *Confidence interval for the mean difference. The significance level used in *t*-test was 0.05.

**Table 3:** Comparison of food groups intake at baseline and end of dietetic intervention.

	Sample size *n* = 101							
	Daily servings				Days/week			
	Baseline	End of intervention	95% CI*	*P* value	Baseline	End of intervention	95% CI*	*P* value
Fruits	1.31 ± 0.89	1.66 ± 0.96	(0.087, 0.606)	0.00	3.34 ± 2.24	4.28 ± 2.43	(0.39, 1.48)	0.00
Vegetables	0.98 ± 0.77	1.15 ± 0.81	(−0.027, 0.374)	0.09	3.05 ± 2.65	3.44 ± 2.51	(−0.13, 0.92)	0.14
Whole grains	9.92 ± 3.67	8.35 ± 2.88	(−2.33, −0.797)	0.00	6.95 ± 0.35	6.97 ± 0.22	(−0.06, 0.10)	0.64
Beans and peas	1.97 ± 1.25	1.09 ± 0.40	(−1.12, −0.64)	0.00	5.21 ± 2.15	4.49 ± 2.36	(−1.23, −0.20)	0.00
Meats								
Fish/poultry	2.99 ± 1.62	2.62 ± 1.14	(−0.73, 0.00)	0.05	3.76 ± 2.15	4.54 ± 2.25	(0.25, 1.31)	0.00
Processed meats	3.13 ± 1.43	2.188 ± 1.04	(−1.30, −0.58)	0.00	5.6 ± 1.75	4.37 ± 2.10	(−1.72, −0.73)	0.00
Dairy								
Cheese	1.911 ± 1.23	1.71 ± 1.13	(−0.52, 0.13)	0.24	2.9 ± 2.25	2.6 ± 2.04	(−0.84, 0.24)	0.28
Whole fat milk	1.72 ± 1.15	1.23 ± 0.62	(−0.76, −0.30)	0.00	5.38 ± 2.47	5.23 ± 2.55	(−0.68, 0.38)	0.58
Fats								
Oils	3.76 ± 2.04	1.39 ± 0.74	(−2.78, −1.95)	0.00	6.68 ± 1.33	6.98 ± 0.19	(0.03, 0.56)	0.03
Saturated fat	1.47 ± 1.08	0.777 ± 0.79	(−0.96, −0.42)	0.00	2.19 ± 2.18	1.1 ± 1.36	(−1.61, −0.56)	0.00
Sweets								
Sweets	1.85 ± 1.29	1.35 ± 1.75	(−0.92, −0.06)	0.02	3.02 ± 2.58	2.2 ± 2.86	(−1.58, −0.04)	0.03
Sugar sweetened beverages	2.79 ± 1.99	1.42 ± 1.21	(−1.78, −0.96)	0.00	6.21 ± 1.72	3.89 ± 2.80	(−2.98, −1.67)	0.00
Fast food								
Fast food meals	0.96 ± 1.41	0.629 ± 0.57	(−0.61, −0.05)	0.02	0.613 ± 0.79	0.703 ± 0.70	(−0.09, 0.26)	0.32
Chips, French fries	1.13 ± 1.15	0.68 ± 0.56	(−0.69, −0.20)	0.00	2.69 ± 2.47	1.36 ± 1.62	(−1.87, −0.78)	0.00
Desserts, refined grain bakery	1.99 ± 1.54	1.32 ± 1.59	(−1.07, −0.25)	0.00	2.85 ± 2.54	1.57 ± 2.20	(−1.90, −0.65)	0.00
Water	2.98 ± 2.02	4.91 ± 2.37	(1.43, 2.43)	0.00	6.615 ± 2.03	6.87 ± 0.91	(0.29, 1.12)	0.00

Data represent mean ± standard deviation. *Confidence interval for the mean difference. The significance level used in paired *t*-test was 0.05.

**Table 4:** Gender comparison of the mean difference of food groups intake after dietetic intervention.

	Daily servings				Days/week			
	Boys (*n* = 55)	Girls (*n* = 46)	95% CI*	*P* value	Boys (*n* = 55)	Girls (*n* = 46)	95% CI*	*P* value
Fruits	0.73 ± 2.75	1.2 ± 2.78	(−1.56, 0.62)	0.40	0.29 ± 1.51	0.41 ± 1.05	(−0.64, 0.40)	0.65
Vegetables	0.27 ± 2.76	0.54 ± 2.64	(−1.34, 0.80)	0.62	−0.02 ± 1.11	0.40 ± 0.85	(−0.81, −0.02)	0.04
Whole grains	0.00 ± 0.38	0.04 ± 0.46	(−0.21, 0.12)	0.61	−1.55 ± 4.25	−1.59 ± 3.46	(−1.50, 1.59)	0.96
Beans and peas	−0.82 ± 2.55	−0.61 ± 2.71	(−1.25, 0.83)	0.69	−0.88 ± 1.16	−0.89 ± 1.30	(−0.47, 0.49)	0.97
Meats								
Fish/poultry	0.56 ± 2.75	1.04 ± 2.63	(−1.55, 0.59)	0.38	−0.29 ± 2.08	−0.46 ± 1.63	(−0.56, 0.89)	0.66
Processed meats	−0.96 ± 2.61	−1.54 ± 2.34	(−0.40, 1.56)	0.25	−0.93 ± 1.79	−0.95 ± 1.89	(−0.70, 0.74)	0.96
Dairy								
Cheese	−0.11 ± 2.88	−0.52 ± 2.62	(−0.685, 1.51)	0.46	−0.31 ± 1.87	−0.07 ± 1.41	(−0.89, 0.40)	0.46
Whole fat milk	−0.31 ± 2.64	0.04 ± 2.76	(−1.42, 0.71)	0.52	−0.66 ± 1.36	−0.38 ± 0.85	(−0.72, 0.16)	0.21
Fats								
Oils	0.03 ± 0.60	0.61 ± 1.86	(−1.14, 0.00)	0.05	−2.59 ± 1.76	−2.11 ± 2.43	(−1.31, 0.34)	0.25
Saturated fat	−1.06 ± 3.08	−1.12 ± 2.04	(−0.96, 1.07)	0.91	−0.53 ± 1.44	−0.9 ± 1.26	(−0.15, 0.90)	0.17
Sweets								
Sweets	−1.15 ± 4.18	−0.41 ± 3.51	(−2.27, 0.81)	0.35	−0.36 ± 2.28	−0.65 ± 2.06	(−0.57, 1.15)	0.51
Sugar sweetened beverages	−2.13 ± 3.30	−2.57 ± 3.36	(−0.88, 1.75)	0.51	−1.53 ± 2.28	−1.18 ± 1.81	(−1.16, 0.48)	0.41
Fast food								
Fast food meals	0.11 ± 0.79	0.07 ± 1.04	(−0.31, 0.40)	0.81	−0.46 ± 1.54	−0.17 ± 1.25	(−0.85, 0.27)	0.31
Chips, French fries	−1.02 ± 2.59	−1.7 ± 2.92	(−0.41, 1.76)	0.22	−0.44 ± 1.32	−0.47 ± 1.19	(−0.46, 0.53)	0.90
Desserts, refined grain bakery	−1.25 ± 3.01	−1.3 ± 3.37	(−1.21, 1.31)	0.94	−0.23 ± 1.91	−1.18 ± 2.19	(0.14, 1.76)	0.02
Water	0.36 ± 1.75	1.13 ± 2.41	(−1.61, 0.08)	0.08	2.00 ± 2.46	1.85 ± 2.65	(−0.85, 1.16)	0.77

Data represent mean ± standard deviation. *Confidence interval for the mean difference. The significance level used in *t*-test was 0.05.
